# Steroid Profiling as an Additional Tool to Confirm One-Sided Hormone Overproduction in Primary Aldosteronism: A Case Report

**DOI:** 10.3389/fendo.2019.00597

**Published:** 2019-08-28

**Authors:** Apostolos Chatzitomaris, Graeme Eisenhofer, Tracy Ann Williams, Otari Worms, Volkmar Nicolas, Martin Reincke, Harald H. Klein

**Affiliations:** ^1^Medical Department I, Endocrinology and Diabetology, Gastroenterology and Hepatology, Bergmannsheil University Hospital, Ruhr University of Bochum, Bochum, Germany; ^2^Institute of Clinical Chemistry and Laboratory Medicine, Technische Universität Dresden, Dresden, Germany; ^3^Division of Internal Medicine and Hypertension, Department of Medical Sciences, University of Turin, Turin, Italy; ^4^Medizinische Klinik und Poliklinik IV, Klinikum der Ludwig-Maximilians-Universität München, Munich, Germany; ^5^Institute of Diagnostic Radiology, Interventional Radiology and Nuclear Medicine, Bergmannsheil University Hospitals, Ruhr University of Bochum, Bochum, Germany

**Keywords:** primary aldosteronism, adrenal venous sampling, steroid profiling, aldosterone-producing adenoma, somatic KCNJ5 mutation

## Abstract

Primary aldosteronism (PA) is the leading cause of secondary hypertension. The source of aldosterone hypersecretion is often due to a unilateral aldosterone-producing adenoma, and unilateral laparoscopic adrenalectomy is recommended in such patients. Before surgery, confirmation of unilateral hypersecretion is necessary. This is optimally performed by adrenal venous sampling (AVS). However, AVS is not always successful e.g., due to difficulties in the cannulation of the right adrenal vein. Here we present the case of a 53-year-old female patient with primary aldosteronism, a left-sided adrenal mass and an inconspicuous right adrenal. AVS was performed, but cannulation of the right adrenal vein failed. Therefore, aldosterone hypersecretion also of the right adrenal could not be excluded despite higher aldosterone concentrations in the left renal and adrenal vein. To increase the certainty that the left sided adrenal mass was the source of aldosterone hypersecretion, steroid profiling was performed in a sample from the inferior vena cava. This revealed markedly elevated levels of 18-oxocortisol, 18-hydroxycortisol, 11-deoxycorticosterone, and 11-deoxycortisol, a steroid profile that strongly suggested that the left sided adrenal mass was an aldosterone producing adenoma, most likely due to a somatic KCNJ5 mutation. Following unilateral adrenalectomy, CYP11B2 immunohistochemistry, and genetics analysis of the resected adrenal confirmed a solitary aldosterone-producing adenoma with intense aldosterone synthase expression, which harbored a previously described KCNJ5 Phe154Cys mutation. Biochemical and clinical cure was confirmed 6 months postoperatively.

## Background

The reported prevalence of primary aldosteronism in patients with hypertension in the general population is around 6% (1). The prevalence in therapy resistant hypertension is over 15%, making primary aldosteronism the most common cause of endocrine hypertension (2). The source of aldosterone hypersecretion is frequently a unilateral adrenal adenoma (3), and unilateral laparoscopic adrenalectomy is recommended in such patients (4). However, bilateral adrenal hyperplasia, nowadays more frequent than unilateral aldosteronism, has to be excluded before proceeding to surgery. In younger patients (age <35 years) with spontaneous hypokalemia and marked aldosterone excess, the radiologic finding of a unilateral adrenal adenoma is regarded as sufficient proof of an aldosterone producing adenoma. In other patients the currently accepted gold standard for subtyping is AVS, as recommended by guidelines (4). AVS is, however, not always successful. In various studies the success rate of AVS varies between 30 and 96% (5–7). Due to anatomic variation, most AVS failures are due to failures of cannulation of the right adrenal vein (8).

Steroid profiling is an emerging method, facilitated by liquid chromatography with tandem mass spectrometry (LC-MS/MS), that involves measurements of multiple steroids, which in addition to aldosterone may include the hybrid steroids 18-hydroxycortisol and 18-oxocortisol (9). Previous studies have shown that these hybrid steroids are higher in adrenal venous samples from aldosterone-producing adenomas (APA) compared to bilateral adrenal hyperplasia (BAH) (10, 11). Furthermore, measurement of these metabolites could predict the underlying mutation, especially the presence of a somatic KCNJ5 mutation in patients with APA (12). Here we demonstrate a case where such steroid profiling helped to confirm a unilateral adrenal adenoma following unsuccessful cannulation of the right adrenal vein.

## Case Presentation

A 53-year-old female presented in our outpatient clinic because of repeatedly low serum potassium levels (2.3 to 2.6 mmol/l) despite oral potassium supplementation. For more than 10 years she had received antihypertensive treatment, lately with valsartane (160 mg od) and lercanidipine (20 mg od). She was not on diuretic medication, did not eat liquorice and had no diarrhea. Her records revealed that already 5 years before her presentation in our outpatient clinic she had marked hypokalaemia (2.5 to 2.7 mmol/l).

**Figure 1:**
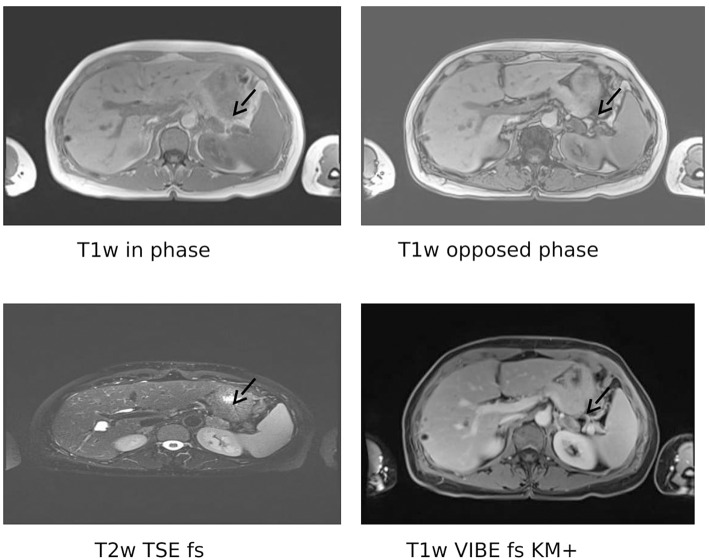
Magnetic resonance imaging. Shown is a left-sided adrenal mass (arrows) with a maximum diameter of 2.3 cm. Only slight signal loss in the opposed phase on Tl weighted-images. Mild contrast-enhancement in the periphery of the tumor. Multiple hepatic cysts. Tlw, T2w: Tl- or T2 weighted, respectively; TSE, turbo spin echo; VIBE, volumetric interpolated breath-hold examination; FS, fat saturated; KM+, with contrast agent.

We detected a suppressed plasma renin concentration (1 ng/l; reference intervals 1.68–23.9 ng/l) and an elevated plasma aldosterone (874 pg/ml; reference intervals 17.6–232 pg/ml), resulting in an aldosterone-to-renin ratio of 874 (upper cut-off of reference intervals in our laboratory <17.5). After correcting the hypokalaemia and switching antihypertensive therapy to doxazosine and lercanidipine, a seated saline infusion test (2 l 0.9% over 4 h) was performed. Plasma aldosterone concentrations were not sufficiently suppressed (0 min: 1,070 pg/ml; 240 min: 731 pg/ml; cut off <50–100 pg/ml) (13), confirming the diagnosis of primary aldosteronism. In a posture test, plasma aldosterone concentrations did not change. An adrenal MRI demonstrated a normal looking right adrenal and a left-sided adrenal mass with a maximal diameter of 23 mm, compatible with an adrenal adenoma (Figure 1).

Options of surgery or long-term mineralocorticoid receptor antagonist therapy were discussed with the patient. We recommended surgical resection of the adenoma as the best option, this dependent on the confirmation that it was the source of the aldosterone excess. Since the patient was not principally opposed, AVS was conducted to exclude a potential contralateral aldosterone hypersecretion (Table 1). The adrenal to peripheral vein (inferior vena cava) cortisol ratios were 1.72 and 10.72 on the right and left side, respectively. With a cut off under cosyntropin stimulation of >3 (14), this indicated that only the left adrenal vein had been properly cannulated. The aldosterone concentration and aldosterone to cortisol ratio was clearly highest in the left adrenal, and also higher at all left renal vein locations compared to those on the right side. Although this confirmed hypersecretion by the left adrenal harboring the adenoma, the failure to properly cannulate the right adrenal vein left the possibility that there was also hypersecretion on the right side (15).

**Table 1:** Adrenal venous sampling.

**Localization**	**Aldosterone (pg/ml)**	**Cortisol (μg/l)**	**Aldosterone/Cortisol**
Inferior vena cava	1,555	337	4.61
Hepatic vein	577	340	1.70
Right renal vein ostium	1,165	276	4.22
Right renal vein distal	1,375	327	4.21
Right renal vein middle	1,355	336	4.03
Right adrenal vein	1,815	579	3.14
Left renal vein ostium	4,260	596	7.15
Left renal vein distal	1,740	295	5.90
Left renal vein middle	4,840	433	11.18
Left adrenal vein	63,600	3,614	17.60

*Continuous cosyntropin (ACTH) stimulation (50 mcg per hour) was started 30 min before and continued throughout sampling from the indicated veins. Shown are the respective aldosterone and cortisol levels as well the aldosterone to cortisol ratio*.

**Figure 2:**
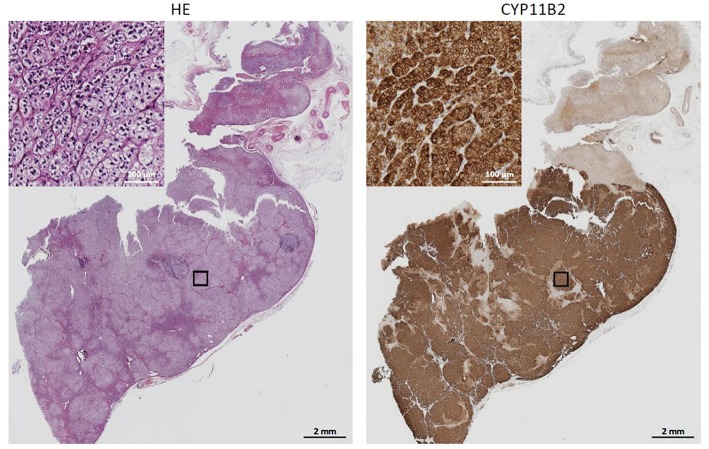
Immunhistochemistry. The formalin-fixed paraffin-embedded adrenal corresponding to the adenoma was stained with HE **(Left)** or subjected to CYP11B2 immunostaining **(Right)**. Demonstrated is an intense CYP11B2 expression.

At this point we looked for a further non-invasive possibility to discriminate between APA and BAH, and therefore performed steroid profiling of a remaining sample taken from the inferior vena cava. Since the patient was in no case willing to have a second AVS, we also started mineralocorticoid receptor antagonist therapy to treat hypertension and hypokalaemia as well as to prepare her for surgery. The steroid profiling revealed markedly elevated plasma concentrations of 18-oxocortisol, 18-hydroxycortisol, 11-deoxycorticosterone, and 11-deoxycortisol (Table 2), a constellation strongly indicating a somatic KCNJ5 mutation in an aldosterone producing adenoma (10, 11). Although initially reluctant with respect to surgery, following these steroid profiling results and convincing her that there now was little doubt that the adenoma of the left adrenal was the source of aldosterone overproduction, the patient underwent left-sided unilateral laparoscopic adrenalectomy. The expected diagnosis of an aldosterone-producing adenoma was confirmed by CYP11B2 (aldosterone synthase) immunohistochemistry (Figure 2). Moreover, direct Sanger sequencing of genomic DNA extracted from 10 μm sections of the formalin-fixed paraffin-embedded adrenal tissue corresponding to the adenoma revealed a heterozygous missense mutation of the KCNJ5 gene (c.861T>G, p.Phe154Cys; data not shown), described previously by Scholl et al. (16).

**Table 2:** Plasma steroid profiles from a blood sample obtained from the inferior vena cava as determined by liquid chromatography with tandem mass spectrometry (LC-MS/MS).

**Steroid**	**Concentration (nmol/l)**	**Reference intervals^a^ (nmol/l)**
18-Oxocortisol^***^	1.75	<0.09
18-Hydroxycortisol^***^	21.0	0.4–3.4
Aldosterone^**^	2.36	0.02–0.67
11-Deoxycorticosterone^**^	3.90	<0.34
11-Deoxycortisol^*^	9.90	0.11–0.69
Corticosterone^*^	61.8	1.6–20.8
21-Deoxycortisol^*^	0.73	<0.22
Cortisol	720	121–700
Cortisone	40.8	25.0–69.9
Androstendione	5.55	0.83–5.62
DHEA	26.2	2.2–22.2
DHEAS	2912	911–6816
17OH-Progesterone	6.2	0.2–6.8
Testosterone	1.54	0.30–2.30
Pregnenolone	19.1	1.1–10.1

The presence of an APA due to a KCNJ5 mutation was indicated from the pattern of increased plasma concentrations of steroids compared to previously reported sex and/or age specific reference intervals (11).

a,*** indicates a steroid characteristically more substantially increased in unilateral APAs due to KCNJ5 mutations than in other unilateral APAs without KCNJ5 mutations or bilateral forms of primary aldosteronism;

** indicates a steroid usually increased more substantially in unilateral APAs than bilateral primary aldosteronism;

* *indicates a steroid often increased in all subtypes of primary aldosteronism compared to primary hypertensive populations*.

Following adrenalectomy spironolactone therapy was terminated. Blood pressure 3 weeks later was 132/86 mm Hg without any antihypertensive medication, potassium was 4.8 mmol/l (3.5–5.5), and the aldosterone/renin ratio was 2.14 (<20). Her primary care physician confirmed that 6 months post surgery her blood pressure was consistently <140/90 mm Hg without medication, and that her potassium concentration remained in the normal range without any supplementation. According to the PASO criteria this is a complete clinical and biochemical success (17).

## Discussion

The finding of unilateral radiological features consistent with an adrenal cortical adenoma is not regarded as sufficient proof for a unilateral aldosterone producing adenoma in patients that are older than 35 years (18). AVS is therefore recommended, with lateralization of aldosterone hypersecretion commonly assessed by calculation of the lateralization index using the higher ratio of aldosterone to cortisol in one vein compared to the other (9). It is an invasive procedure, and even if performed by an experienced radiologist in specific centers it is, as in the present case, not always successful. Here we demonstrate that in such situations steroid profiling can help by providing evidence for the existence of an aldosterone hypersecreting adenoma.

It has been previously shown that patterns of steroid expression in APA are dependent of the underlying somatic mutations (12), and in most cases differ from the pattern seen in BAH (10, 11). In the present case, the substantially increased levels of 18-oxocortisol and 18-hydroxycortisol in the vena cava sample left little doubt that an APA with a KCNJ5-mutation and not BAH caused the aldosteronism, and thus helped us with the decision to remove the left sided adenoma despite inconclusive AVS. The presence of the KCNJ5 mutation was later confirmed in the biopsy. Due to the lack of material we did not perform steroid profiling in the adrenal or renal veins. Since the aldosterone concentration and aldosterone/cortisol ratio in the left adrenal vein clearly demonstrated hypersecretion on this side and the steroid profile derived from the inferior vena cava was so clearly suggestive of an APA, this would, however, have added little information in the present case.

Steroid profiling through (LC-MS/MS) is non-invasive and requires only a minimal volume of blood. On the other hand, instrumentation is expensive and requires a high level of expertise, with so far no available prospective data (9). Potentially, steroid profiling will in the future be a non-invasive tool that can help to avoid AVS, at least in certain presentations of patients with primary aldosteronism.

## Consent

Written informed consent was obtained from the patient for the genetic examination and the publication of this report and any accompanying images.

## Ethics Statement

(Translation from German): I agree within, that a case report of my disease will be published, obviously without any credits or hints concerning my person. That concerns also the figures (MR-images, blood results, immunohistochemistry of the obtained materials or sequencing analysis in the obtained material).

## Author Contributions

VN was the radiologist who conducted the MRI and performed the AVS. GE was responsible for the steroid profiling. TW performed histopathologic analyses and DNA sequencing. MR gave the impulse to initiate steroid profiling. AC, OW, and HK were the treating endocrinologists. All authors read and approved the final manuscript.

### Conflict of Interest Statement

The authors declare that the research was conducted in the absence of any commercial or financial relationships that could be construed as a potential conflict of interest.
